# Macular edema after rhegmatogenous retinal detachment repair: risk factors, OCT analysis, and treatment responses

**DOI:** 10.1186/s40942-020-00254-9

**Published:** 2021-01-25

**Authors:** Cameron Pole, Ismael Chehaibou, Andrea Govetto, Sean Garrity, Steven D. Schwartz, Jean-Pierre Hubschman

**Affiliations:** 1 Retina Division, Stein Eye Institute, University of California Los Angeles, 100 Stein Plaza, Los Angeles, CA 90095-7002 USA; 2 Ophthalmology Department, AP-HP, Hôpital Lariboisière, Université de Paris, 75010 Paris, France; 3 Ophthalmology Department, Fatebenefratelli-Oftalmico Hospital, ASST-Fatebenefratelli-Sacco, Milan, Italy; 4 Tufts Medical Center/New England Eye Center, Ophthalmic Consultants of Boston, Boston, MA USA

**Keywords:** Intravitreal injection, Macular edema, Retinal detachment, Spectral-domain optical coherence tomography, Vitrectomy, Corticosteroids

## Abstract

**Purpose:**

To investigate risk factors, imaging characteristics, and treatment responses of cystoid macular edema (CME) after rhegmatogenous retinal detachment (RRD) repair.

**Methods:**

Consecutive, retrospective case–control series of patients who underwent pars plana vitrectomy (PPV) and/or scleral buckling (SB) for RRD, with at least six months of follow-up. Clinical and surgical parameters of patients with and without CME (nCME), based on spectral-domain optical coherence tomography (OCT), were compared.

**Results:**

Of 99 eyes enrolled, 25 had CME while 74 had nCME. Patients with CME underwent greater numbers of surgeries (*P* < 0.0001). After adjusting for number of surgeries, macula-off RRD (*P* = 0.06), proliferative vitreoretinopathy (PVR) (*P *= 0.09), surgical approach (PPV and/or SB, *P *= 0.21), and tamponade type (*P *= 0.10) were not statistically significant, although they all achieved significance on univariate analysis (*P* = 0.001 or less). Intraoperative retinectomy (*P* = 0.009) and postoperative pseudophakia or aphakia (*P *= 0.008) were more frequent in the CME group, even after adjustment. Characteristics of cCME on OCT included diffuse distribution, confluent cysts, and absence of subretinal fluid or intraretinal hyperreflective foci. Macular thickness improved significantly with intravitreal triamcinolone (*P *= 0.016), but not with anti-vascular endothelial growth factor agents (*P* = 0.828) or dexamethasone implant (*P *= 0.125). After adjusting for number of surgeries and macular detachment, final visual acuities remained significantly lower in the CME vs nCME group (*P *= 0.012).

**Conclusion:**

Risk factors of CME include complex retinal detachment repairs requiring multiple surgeries, and pseudophakic or aphakic lens status. Although this cCME was associated with poor therapeutic response, corticosteroids were the most effective studied treatments.

## Background

Cystoid macular edema (CME) is a common retinal condition characterized by macular thickening with intra-retinal fluid accumulation, often accompanied by decreased visual acuity (VA) [1]. It may develop as a complication of a wide spectrum of retinal diseases including diabetic retinopathy (DR), uveitis, exudative age-related macular degeneration (AMD), retinal vein occlusion (RVO), and genetic syndromes such as retinitis pigmentosa (RP) [2].

Although the pathophysiology of CME is multifactorial, breakdown of the inner blood retinal barrier is a common endpoint in most cases [1]. Current theories suggest subclinical inflammation as responsible for post-rhegmatogenous retinal detachment (RRD) CME [3]. While progressive leakage may be outlined with fluorescein angiography (FA) as the *gold*-*standard* for CME diagnosis, optical coherence tomography (OCT) is currently the most common imaging modality in the diagnosis and characterization of CME, as it is non-invasive and provides high resolution cross-sectional imaging of retinal anatomy [4], allowing easier and more frequent follow-up,

Rhegmatogenous retinal detachment is characterized by progressive accumulation of subretinal fluid due to retinal breaks. Although surgical repairs, including scleral buckle (SB) and pars plana vitrectomy (PPV), are effective surgical treatments, some cases with successful reattachment may have poor visual outcomes related to postoperative CME development [5, 6], which may persist for years in a minority of patients [7]. Retrospective and observational studies using FA and OCT have shown rates of post-vitrectomy CME varying from 5.5% after PPV for symptomatic floaters to 40% after complicated detachment repairs [6, 8, 9]. Treatments for CME primarily target inflammatory and pro-angiogenic mediators, but standard therapies such as anti-vascular endothelial growth factor (anti-VEGF) therapies may be ineffective for post-RRD CME [9, 10].

There is little data on post-RRD CME risk factors, rates, and anatomical characteristics [3, 5, 11]. Therefore, this observational study was designed to compare a consecutive case series of eyes with *versus* without post-RRD CME, with the aim to determine its risk factors and describe its clinical characteristics and therapeutic outcomes.

## Methods

This was a retrospective, observational study approved by the medical center’s institutional review board, University of California Los Angeles Office of Human Research Protection (IRB#16-000574). This study adhered to the tenets of the Declaration of Helsinki and the rules of the Health Insurance Portability and Accountability Act of 1996.

Electronic health records (EHR) from a large academic referral center (Stein Eye Institute at UCLA) were reviewed. Current Procedural Terminology (CPT) coding records of surgical procedures from January 2015 to December 2017 were queried.

### Population

All candidates underwent SB, PPV, or combined procedures for RRD, performed by two experienced vitreoretinal surgeons (JPH and SDS), with at least 6 months of follow up after surgery. Records were evaluated through July 2018.

Exclusion criteria were severe ocular trauma, uveitis, DR, endophthalmitis, RVO, myopic retinoschisis, or advanced dry or wet AMD.

### Spectral Domain-OCT Analysis

All patients diagnosed with CME were examined with eye-tracked OCT. All OCTs were acquired with the Spectralis^®^ (Heidelberg Engineering GmbH, Heidelberg, Germany) and RS-3000 (Nidek^®^ Inc, San Jose, CA) devices. All CME was analyzed with Spectralis^®^ OCTs consisting of 19 horizontal B-scans and manually adjusted for foveal centration. All OCT scans were carefully reviewed independently by two graders (CP, JPH) on the Heidelberg Eye Explorer software (Version 1.10.0.0).

A diagnosis of CME was noted if intraretinal hyporeflective spaces were noted in the inner nuclear layer (INL) and/or outer plexiform layer (OPL). Retinal thickness measurements were not used for CME diagnosis, as eyes had varying levels of atrophy.

Eyes were classified as having postoperative transient CME (tCME), chronic CME (cCME), or no CME (nCME). Both tCME and cCME were included as all CME (aCME) for statistical analysis. Postoperative tCME was defined as CME seen on OCT within 6 months of the final RRD, lasting less than 6months, and resolving using topical treatment. Postoperative cCME was defined as CME seen on two OCTs at least 6 months apart, based on previous reports [12].

Recorded characteristics of cCME on OCTs included presence of subretinal fluid, layers of CME involvement, presence of intraretinal hyperreflective foci, and integrity of outer retinal layers. Efficacy of anti-VEGF, triamcinolone acetonide (TA), or dexamethasone implant (Ozurdex®, Allergan Inc, Irvine, California) (DEX) injections were assessed after 4–6 weeks, if OCT was available. To determine treatment effect, pre- and post-injection OCTs were analyzed for central subfield thicknesses (CST) and inner macular volumes, comprised of the central five areas of the standard early treatment for diabetic retinopathy study (ETDRS) subfields [13].

### Clinical charts analysis

Preoperative RRD parameters, intraoperative and post-operative data were collected. Glaucoma was counted if the patient carried this diagnose from a glaucoma specialist. Visual acuity was measured on a Snellen chart and converted to logarithm of the minimum angle of resolution (LogMAR) values for statistical analysis. Count fingers and hand motions vision were recorded as 1.98 and 2.28 LogMAR, respectively, based on previous studies using the Freiburg Visual Acuity Test [14]. Type of cCME treatment and number of intravitreal injections were included.

### Statistical analysis

Qualitative values were listed as ratios and percentages while quantitative values were presented as mean ± standard deviation (SD). Qualitative variables were compared using the Fisher exact test. To compare continuous data between two groups, a Mann–Whitney U test was used. The Wilcoxon signed rank test was used to analyze changes in CST and inner retinal volume. The Kruskal–Wallis test was used to compare pre-injection OCT parameters between groups. The Shapiro–Wilk test assessed the normality of variable distribution. Covariate adjusted differences between CME groups were assessed using regression modeling (i.e. logistic, linear, and multinomial) using the number of surgeries as the covariate. Final visual acuity (logMAR) was log transformed in multivariable analyses and used the additional covariate of macula on/off. All statistics were performed in Stata SE 15.1 (StataCorp LP, College Station, TX). A *P* value of less than 0.05 was considered statistically significant. Denominators of ratios were less than the total number of eyes in the category if eyes could not be included in analyses due to missing or incomplete records.

## Results

### Population

**Fig. 1:**
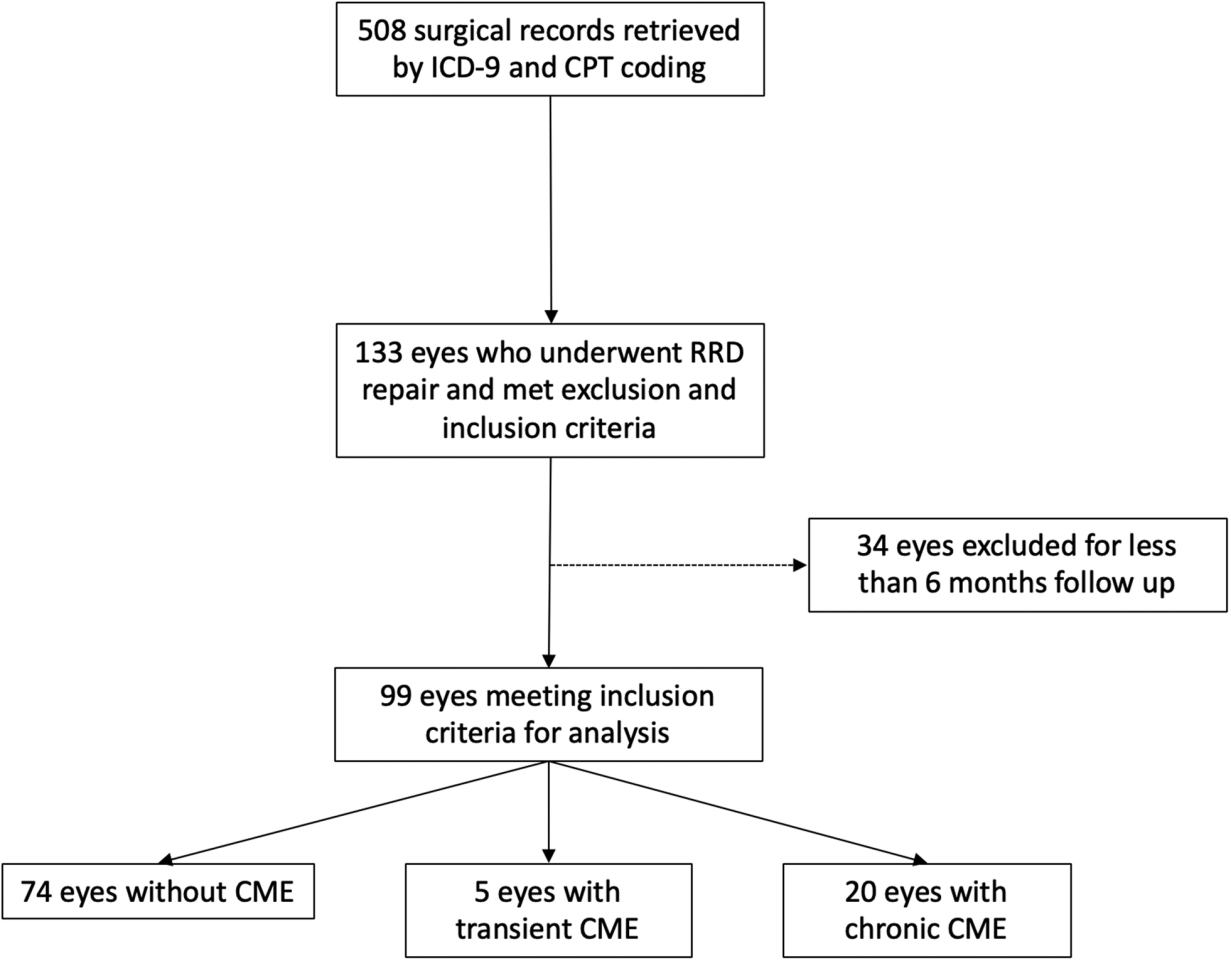
Flowchart of patient selection process. ICD-9: International Classification of Disease, 9th edition. *CPT* Current Procedural Terminology, *CME* Cystoid Macular Edema

A flowchart of population selection is shown in Fig. 1. A total of 508 surgical records were retrieved using CPT codes from January 2015 to December 2017. Of these, 133 eyes undergoing RRD repair met inclusion and exclusion criteria. Of these, 34 had less than 6 months of follow-up. The remaining 99 eyes of 97 patients were included for analysis. Of these, 20 patients (20%) had cCME, 5 (5%) had tCME, and 74 (75%) had nCME. Our primary analyses examine tCME and cCME as a single group, all CME (aCME), in comparison to nCME due to the small sample size for tCME. Descriptive statistics for all three groups can be found in the Additional file 1: Table S1.

### CME risk factors

Demographic and surgical data are summarized by CME group in Table 1. There was no difference in age at last surgery between patients in the aCME group (64.1 ± 11.6 years) *versus* patients in the nCME group (56.7 ± 18.0 years, *P* = 0.092). There was no significant difference in gender (*P* = 0.093), glaucoma status (*P* = 0.258), or length of follow-up (*P* = 0.869). Among those with glaucoma, there was no difference in the rates of topical prostaglandin analogs, other topical medications, or glaucoma surgery between groups (*P* = 0.992).

**Table 1:** Demographics, baseline characteristics, and surgical data of patients with aCME and nCME

	aCME	nCME	*P* value	Adjusted *P* value^1^
Demographic data				
Number of eyes	25 (25%)	74 (75%)		
Follow-up (months)	21.4 ± 12.1	20.4 ± 10.8	0.87	
Sex, Female	8 (32%)	38 (34%)	0.09	
Age (years)	64.1 ± 11.6	56.7 ± 18.0	0.09	
Clinical data				
Right eye	11 (44%)	40 (54%)	0.38	
Glaucoma	4 (16%)	6 (8%)	0.26	
Lens status			< 0.001	0.008
Phakic	1 (4%)	44 (60%)		
Pseudophakic	14 (56%)	28 (38%)		
Aphakic	10 (40%)	2 (3%)		
Macula off^a^	20/24 (83%)	31/70 (44%)	0.001	0.06
PVR Stage C^a^	15/24 (63%)	5/74 (7%)	< 0.001	0.09
Final VA (LogMAR)	0.85 ± 0.80	0.20 ± 0.30	< 0.001	0.012^b^
ERM	18 (72%)	28 (38%)	0.005	
Surgical details				
Number of surgeries	3.5 ± 1.8	1.4 ± 0.9	< 0.001	
Multiple PPV	21 (84%)	17 (23%)	< 0.001	–^c^
Referred after surgery elsewhere	12 (48%)	5 (7%)	< 0.001	0.31
Number of surgery outside	1 ± 1.3	0.095 ± 0.4	< 0.001	
Type of surgery			< 0.001	0.21
SB	1 (4%)	25 (34%)		
PPV	7 (28%)	28 (38%)		
PPV + SB	17 (68%)	21 (28%)		
Tamponade agent			< 0.001	0.10
None/Air	1 (4%)	24 (32%)		
Gas (SF_6_ or C_3_F_8_)	8 (32%)	46 (62%)		
Silicone oil	16 (64%)	4 (5%)		
Cryotherapy^a^	4/24 (17%)	30/73 (41%)	0.047	0.036
Retinectomy	9 (36%)	4 (5%)	< 0.001	0.009
PFCL^a^	18/23 (78%)	35/47 (75%)	0.73	0.38

*aCME* all (chronic + transient) cystoid macular edema, *nCME* no cystoid macular edema, *PVR* proliferative vitreoretinopathy, *VA* visual acuity, *LogMAR* (logarithm of the minimum angle of resolution), *PPV* pars-plana vitrectomy, *ERM* epiretinal membrane, *SB* scleral buckle, *PFCL* perfluorocarbon liquid

^a^Denominators are provided if the number is less than the total number of eyes in the category due to missing or incomplete data

^b^Final VA adjusted P value from a model with covariates for total number of surgeries and Macula on/off

^c^Adjusted model not possible due to collinearity of Multiple PPV with number of surgeries (i.e. those with Multiple PPV had greater than 2 surgeries, while those with no PPV had fewer)

^1^P-value for difference after adjustment for total number of surgeries

Eyes in the aCME group underwent a significantly greater number of retinal surgeries (3.5 ± 1.8) compared with eyes in the nCME group (1.4 ± 1.9) *(P* < 0.001). Due to the high collinearity between CME status and number of surgeries, multivariate analysis using this as a covariate was performed. Final lens status differed significantly between groups after adjustment (*P *= 0.008), with only one eye in the aCME group remaining phakic. A higher rate of aCME eyes had a macula-off retinal detachment (20/24, 83%), compared with nCME eyes (31/70, 44%, *P *= 0.001). Proliferative vitreoretinopathy (PVR) stage C was more frequent in the aCME group (15/24, 63%) *versus* the nCME group (5/74, 7%), *P* < 0.0001. However, both macula-off status (*P *= 0.06) and presence of PVR C (*P *= 0.09) lost statistical significance after adjustment for the total number of surgeries performed. Surgical approaches were statistically different between the aCME and nCME groups: primary SB in 1/25 (4%) aCME eyes vs. 25/74 (34%) nCME eyes, PPV in 7/25 (28%) aCME eyes vs. 28/74 (38%) nCME eyes, and combined SB + PPV in 17/25 (68%) aCME eyes vs. 21/74 (28%) nCME eyes (*P *<0.0001). However, these differences in the surgical approach were not reliably different after adjustment for the number of surgeries. Rates of retinectomy were higher in the aCME group than the nCME group after adjustment (9/25, 36% vs 4/74, 5%, *P* = 0.009). Rates of cryotherapy were higher in the nCME group (30/74, 41%) than aCME group (4/24, 17%), even after adjustment (*P *= 0.036). Unadjusted differences in tamponade agent between groups were statistically significant (P < 0.0001). Notably, 16 out of 25 (64%) aCME eyes received silicone oil (SO) at least once, while only 4 out of 74 (5%) of nCME eyes did. However, tamponade differences were no long significant after covariate adjustment. There was no difference in the use of perfluorocarbon liquid (PFCL) (*P* = 0.728).

At last examination, VA was significantly lower in aCME group (0.85 ± 0.80 LogMAR) than in nCME group (0.20 ± 0.30 LogMAR)*, P* < 0.0001. When adjusting for the number of surgeries and macular detachment, the marginal estimates for between group differences in LogMAR were attenuated (aCME = 0.55 vs nCME = 0.26), though still statistically significant (*P *= 0.012).

Two patients had non-simultaneous RRDs in each eye. One patient was 23 years of age at the time of both surgeries and underwent SB with cryotherapy in each eye for inferior chronic RRD, without CME development. The other patient was 83 at the time of final surgery in both eyes, had initial surgeries performed elsewhere, had multiple PPVs in both eyes, and received SO in both eyes, and this patient developed cCME in both eye.

### OCT characteristics of cCME

**Fig. 2:**
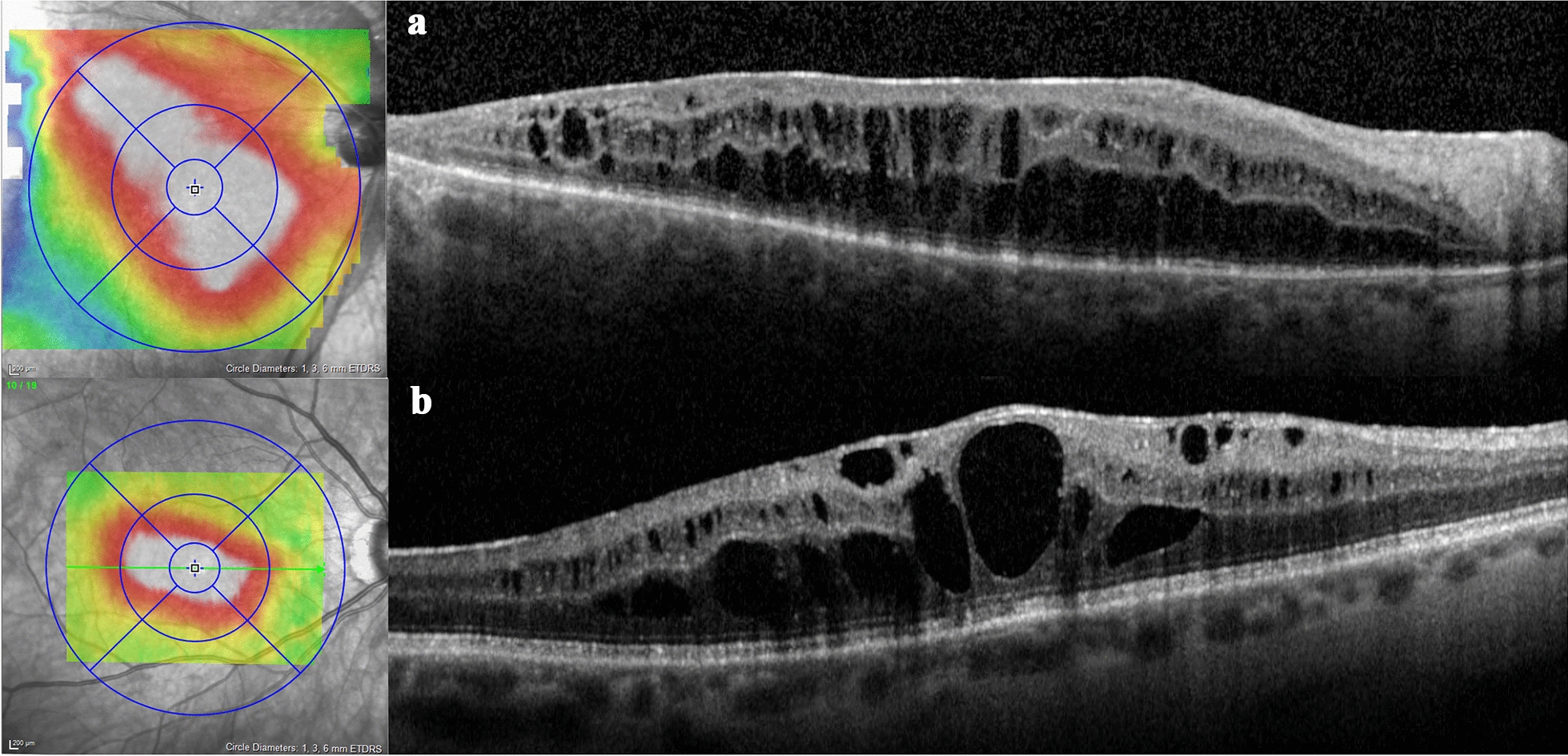
Spectral-domain optical coherence tomography and infrared image elevation overlays of two different patients with chronic cystoid macular edema post-rhegmatogenous retinal detachment. The scan in Row A demonstrates schisis-like changes. The scan in Row B demonstrates confluent cystic cavities spanning retinal layers that developed over two years. In both scans, note diffuse, asymmetric distribution of retinal cysts crossing the horizontal raphe, involvement of inner and outer retinal layers, absence of subretinal fluid, and relative preservation of outer retinal bands subjacent to retinal edema

Eyes in the cCME group (n = 20) shared particular qualities on OCT (Fig. 2). All eyes had diffuse CME involving the four macular quadrants. The CME always involved the fovea but had variable extent into peripheral macula and was often asymmetric. Cysts were uniformly present in the INL and OPL, with occasional ganglion cell layer involvement. Florid CME often assumed a retinoschitic appearance. With time, cysts coalesced into larger confluent cavities with irregular, polygonal shapes. These cysts often spanned within the same retinal layer and across adjacent layers. Temporary resolution of these cysts after treatment disclosed disorganization and variable atrophy of the retinal layers in areas of cyst confluency. If CME recurred after treatment, it typically recurred in the same distribution of the macula.

Outer retinal layer integrity was heterogeneous. On the first OCT with CME after the final RRD repair, ellipsoid zone (EZ) disruption was seen in 18 eyes (90%), external limiting membrane (ELM) disruption in 14 eyes (80%), and retinal pigment epithelial (RPE) disruption in 11 eyes (55%). Remarkably, there was no case with subretinal fluid (SRF), and no case of intraretinal hyperreflective foci or hemorrhage.

An epiretinal membrane (ERM) was detectable on OCT during the post-operative follow-up period in 17/20 (85%) cCME eyes, 2/5 (40%) tCME eyes, and 28/74 (38%) of nCME eyes (*P *= 0.005). Evidence of traction on OCT, such as inner retinal wrinkling or ectopic inner foveal layers, was appreciable in only 4 of the 17 cCME eyes with ERM. However, the severity of CME was out of proportion to the ERM changes in all but one of these four eyes.

### CME Treatments

All patients with tCME (n = 5) and cCME (n = 20) received topical medications. Intravitreal injections and surgical interventions were administered according to physician discretion. All patients received corticosteroid drops, non-steroidal anti-inflammatory agent (NSAID) drop, or a combination of both for at least two months after the diagnosis of CME. If the CME failed to respond, patients thereafter received intravitreal injections of anti-VEGF (bevacizumab, ranibizumab, aflibercept), or steroids (triamcinolone acetate (TA), and/or dexamethasone intravitreal implant (DEX)).

**Fig. 3:**
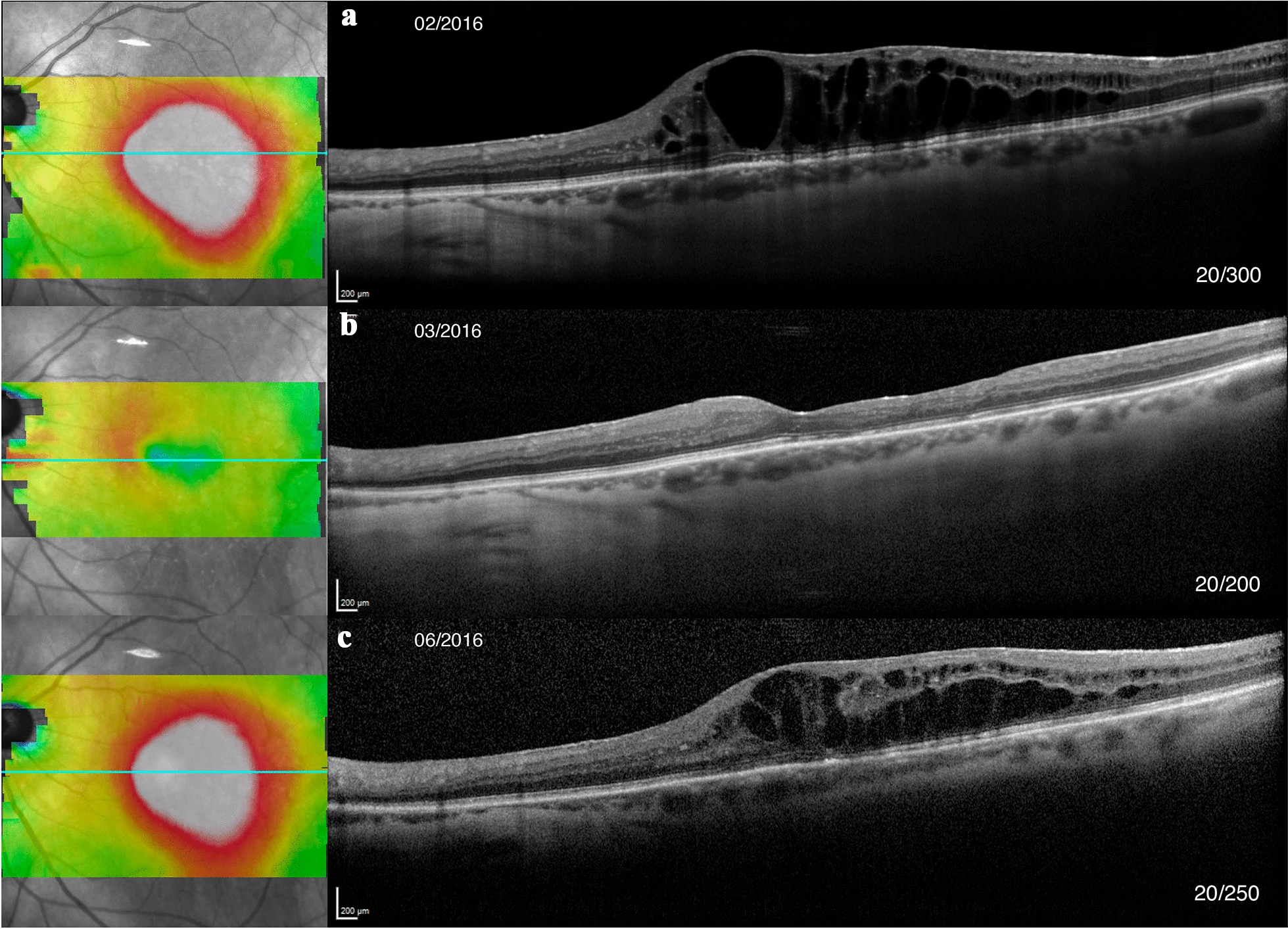
Spectral-domain optical coherence tomography (OCT) images of chronic cystoid macular edema (CME) post-rhegmatogenous retinal detachment (RRD) repair of the left eye, with dates and visual acuities (VA). Panel A: OCT prior to dexamethasone implant (DEX) injection. Panel B: OCT 1 month after DEX injection, showing resolution of CME but retinal layer atrophy. Modest VA improvement was noted. Panel C: OCT four months after injection, showing recurrence of CME in a similar distribution and slight decrease in VA

The five patients (25%) with tCME had permanent resolution of CME with drops. Table 2 summarizes intravitreal treatments and anatomical responses of cCME. Five patients received at least one bevacizumab (Avastin®, Genentech Inc., San Francisco, CA, USA) injection, and one of these patients also received aflibercept (Eylea®, Regeneron Inc., Tarrytown, NY, USA) injections. In cCME eyes, there was a significant CST (*P* = 0.016, Wilcoxon signed rank test) and volume (*P* = 0.016) decrease after TA. (*P* = 0.125) (Fig. 3). There was no difference in pre-injection CST or volume between groups (*P* = 0.397, *P* = 0.457). There was no significant change in CST or volume with anti-VEGF treatment (*P *=0.915, *P* = 0.828) or DEX (*P* = 0.434, *P* = 0.125). No patient developed elevated intraocular pressure (IOP) after intravitreal injection requiring treatment. One patient developed sterile endophthalmitis after her seventh TA injection that spontaneously resolved without sequelae. A PPV for an ERM was performed in 9/16 cCME eyes with OCT evidence of ERM, with full resolution of the CME in only one eye.

**Table 2:** Treatments for chronic cystoid macular edema (cCME) and anatomical responses on spectral-domain optical coherence tomography

Type of treatment	Anti-VEGF	TA	DEX	
Number of eyes	5	7	4	
Number of Injections (Median; [Range])	2.5, 1-14	2.0, 1-10	2.5, 1-7	
CST pre-injection (μm)	401 ± 84.9	481 ± 104	397 ± 57.0	*P *= 0.397
CST post-injection (μm)	393 ± 106	402 ± 102	355 ± 80.4	
Percent CST change (μm)	− 1.44 ± 17.1, *P *= 0.915	− 15.6 ± 16.6, *P *= 0.016	− 11.0 ± 10.7, *P *= 0.434	
Inner macular volume pre-injection (mm^3^)	2.81 ± 0.43	3.18 ± 0.56	3.12 ± 0.80	*P *= 0.457
Inner macular volume post-injection (mm^3^)	2.74 ± 0.53	2.72 ± 0.53	2.66 ± 0.486	
Percent (%) inner macular volume change (mm^3^)	−2.49 ± 12.35, *P *= 0.828	−13.9 ± 10.8, *P* = 0.016	−10.7 ± 25.7, *P* = 0.125	

Values are listed as averages with standard deviations

*VEGF* vascular endothelial growth factor, *TA* triamcinolone acetate, *DEX* dexamethasone implant, *CST* central subfield thickness

## Discussion

Chronic CME after retinal detachment repair remains a challenging complication. In this paper, the risk factors for post-RRD CME, its OCT characteristics, and treatments outcomes are described.

Chronic post-RRD CME is thought to be pathophysiologically distinct from other etiologies of CME [3]. Among CME etiologies such as uveitis, RVO, and DME, many of the cytokines and damaged tissue responses are shared [1, 2, 15]. Certain CME etiologies, however, may have unique pathophysiologic mechanisms despite phenotypic similarities [16]. Entities with a significant pro-angiogenic component, such as exudative AMD, may respond to anti-VEGF agents, while those with a broad inflammatory component, such as uveitic CME or Irvine-Gass syndrome, may respond better to anti-inflammatory drugs [12].

While some studies found no risk factor differences for CME rates [5, 17], some series have, on univariate analyses, reported increased rates in pseudophakic [18] and aphakic eyes [6], older patients, more extensive RRD, and a history of a detached macula. In the present study, lens status was significantly different between groups, with increased pseudophakia and aphakia in aCME eyes. Unicameral communication in vitrectomized eyes modifies circulation of inflammatory cytokines, as animal studies have noted changes in oxygen and antioxidant gradients [19]. Higher rates of pseudophakia/aphakia in the aCME group may be related either to the actual lens surgery or to the complexity of the vitreo-retinal surgeries requiring lens extraction. As a substantial proportion of eyes with complicated RRD will be made pseudophakic or aphakic, anticipating CME in complex cases can have prognostic implications.

Eyes with CME had a greater number of surgeries, higher rates of PVR grade C and retinectomy, and higher rates of SO use. Many studies have shown increased inflammation and CME with more complicated ocular surgeries and inflammatory risk factors [3, 11, 20]. Re-detachments are frequently associated with PVR formation and warrant additional surgeries, both of which can increase intraocular inflammation and possible risk for CME [21]. Retinectomy is helpful when PVR membranes are not amenable to mechanical peeling, and therefore retinectomy likely indicates severe pathology rather than directly causing CME.

Macular detachment was associated with a higher risk of CME, which is in line with prior papers [18]. Of note, previous studies have noted outer nuclear layer CME on OCT of the detached macula [22, 23]. Although the retinal hydration theory, implicated in macular hole edema formation [24], may contribute to post-RRD CME, the presence of leakage on FA suggests dynamic fluid movements as opposed to static, non-leaking cysts. Moreover, absence of SRF after RD repair would theoretically lead to rapid elimination of intraretinal fluid by normal pumping mechanisms. Although such studies for macular detachment and CME development have not been explored [18, 25], permanent damage to retinal cellular elements while detached may lead to persistent dysfunction and contribute to CME.

There was a significant difference in surgical approaches between groups, with higher rates of combined SB and PPV in aCME eyes. This is not surprising, given that scleral buckles are often combined with PPV for complex or recurrent detachments to support the vitreous base and/or areas of retinal pathology. However, there was significantly more cryotherapy in the nCME group. Cryotherapy at our institution is only used during primary scleral buckling, usually for limited and uncomplicated detachments in phakic patients. While data comparing CME rates between PPV and SB are scant, the correlation between more complicated detachments and CME is consistent [3, 11, 18].

After adjusting for the number of surgeries, type of surgery (*P *= 0.21), macular detachment (*P *= 0.06), PVR Grade C (*P* = 0.09) and tamponade type (*P* = 0.10) lost statistical significance. This may be related to the limited sample size, as there remained a trend towards significance. Moreover, these factors are clinically related to the number of surgeries and surgical failure. The interplay of inflammation among these factors requires more formal study.

Characteristics of CME on OCT can be useful diagnostic clues, and post-RRD cCME displays distinguishing OCT features (Fig. 2). Previous studies have examined OCTs of various conditions associated with CME and noted distinctive findings [13]. These findings could then be used to diagnose conditions accurately as well as account for variability in VA [26]. Post-RRD cCME shares features of uveitic CME, such as diffuse macular distribution, inner and outer layer cysts, and absence of hyperreflective foci. This contrasts to post-RRD tCME, which is much less severe, more central and fleeting, and may be a variant of pseudophakic CME.

The presence of ERM is common after RRD and may confound CME diagnosis [16]. Although there was a significant difference between groups in the presence of ERM on OCT, there was resolution of CME in only one eye after ERM peeling, suggesting that traction plays a small role in most cases of post-RRD CME. Therefore, there should be high suspicion for post-RRD cCME in any patient status-post RRD repair that has severe, diffuse CME without SRF in the absence of other typical inflammatory or tractional signs.

The RPE has a well-studied role in pumping syneretic vitreous fluid through the retina and into the choroidal space [1]. Active fluid transport regulation by the RPE and Muller cells along with maintenance of tight junctional proteins are thought to mitigate CME accumulation [1, 2, 15], and dysfunction of these cells causes an imbalance of fluid inflow and egress. Previous papers examining CME OCT findings note varying SRF rates, from 5% in uveitic CME up to 100% in central RVO-associated CME [1, 4, 13, 27]. Therefore, the absence of SRF in cCME suggests a grossly functioning RPE and outer retinal barrier.

Intravitreal corticosteroids were more effective than intravitreal anti-VEGF or topical medications for cCME in our series. Recent investigations have shown success with intravitreal corticosteroids for chronic post-RRD CME [16, 25]. Thanos et al. found favorable responses to DEX all eyes, but in all cases CME recurred after 3 months. This aligns with pharmacokinetic studies showing a dual-phase response of high dexamethasone concentrations for the first 2 months after delivery followed by a precipitous decrease during the third month [28]. Experimental studies have demonstrated a reduced half-life of anti-VEGF agents and triamcinolone acetate in vitrectomized eyes compared with non-vitrectomized eyes [28, 29], but similar clearances between eyes with DEX. Statistically significance for anatomical improvement was not reached for DEX in our series, likely due to the small number of eyes. Moreover, aphakia has been suggested to cause increased unicameral circulation of inflammatory cytokines [6, 30], but aphakia precludes the use of DEX. One randomized controlled trial evaluating PPV with SO for RRD with grade C PVR found a significant decrease in CME occurrence at 6 months post-operatively in those with intraoperative DEX [31]. Corticosteroids have been shown to modulate a number of cytokines secreted by retinal cells, such as tumor necrosis factor-α, interleukins-1β, 6, and 8, as well as induce expression of occludin, ZO-1, and claudin-5 [1, 16, 31]. Steroids also modulate expression of aquaporin, predominantly expressed in end-feet of Müller cells and astrocytes. Corticosteroids may therefore stabilize the BRB and encourage resolution of CME, accounting for the increased efficacy of corticosteroids over anti-VEGF agents. Nevertheless, disadvantages of TA and DEX include accelerated cataract formation and risk of increased IOP; however, most patients with cCME will require cataract extraction, and no patient in our series required treatment for ocular hypertension.

Average final visit VA was significantly worse in the aCME group even after adjusting for macula-off status and number of surgeries. Reports on recalcitrant CME after PPV for RRD, despite anatomic improvement, found only short-term visual acuity gains [16, 25].

Irvine-Gass syndrome (IGS) is another potential diagnosis in these cases. We did not regularly perform FA or optic disc evaluations to check for optic nerve head leakage during the course of follow up. However, IGS is not described after PPV and has been described as a potential treatment option in many cases [32]. Therefore, IGS would have likely responded to topical treatments, steroid injections, or PPV. The OCT appearance of IGS is also less diffuse, more foveocentric, and may be associated with SRF, as opposed to characteristics noted with post-RRD cCME.

Our paper has a relatively large sample size of post-RRD CME, long-term patient records and follow up, and variety of treatments. Despite this, our study has several limitations. The retrospective analysis precluded standardized imaging and treatment protocols. Significant loss to follow-up likely led to underreporting of chronic post-RRD CME and an inability to accurately determine incidence. The high percentage of CME likely relates to inclusion of eyes that had initial RRD repairs prior to the inclusion period and multiple referrals for complex cases. We were unable to determine after which surgery CME appeared due to inconsistent timing and absence of OCT acquisition between surgeries, or missing outside records. A small number of eyes received anti-VEGF injections, and greater numbers may show CME improvement. A larger, prospective study evaluating complex macular surgeries is warranted.

In conclusion, cCME after RRD is a complex entity with interconnected risk factors. A high index of suspicion based on risk factor and imaging characteristics can allow anticipation of cCME development and early treatment. Currently, corticosteroids have the most evidence of treatment success, and prompt intervention may provide better functional and structural outcomes.

## Supplementary information

**Additional file 1: Table S1.** Descriptive statistics for all three groups, as explained in results population section.

## Data Availability

The datasets used and/or analysed during the current study are available from the corresponding author on reasonable request.

## Abbreviations

CME: Cystoid macular edema
tCME: Transient CME
cCME: Chronic CME
aCME: All CME
DR: Diabetic retinopathy
AMD: Age-related macular degeneration
RVO: Retinal vein occlusion
RP: Retinitis pigmentosa
RRD: Rhegmatogenous retinal detachment
FA: Fluorescein angiography
OCT: Optical coherence tomography
SB: Scleral buckle
PPV: Pars plana vitrectomy
VEGF: Vascular endothelial growth factor
EHR: Electronic health records
CPT: Current procedural terminology
TA: Triamcinolone acetonide
DEX: Dexamethasone intravitreal implant
CST: Central subfield thickness
ETDRS: Early treatment for diabetic retinopathy study
SD: Standard deviation
PVR: Proliferative vitreoretinopathy
PFCL: Perfluorocarbon liquid
INL: Inner nuclear layer
OPL: Outer plexiform layer
EZ: Ellipsoid zone
ELM: External limiting membrane
ERM: Epiretinal membrane
RPE: Retinal pigment epithelium
SRF: Subretinal fluid
NSAID: Non-steroidal anti-inflammatory drug
IOP: Intraocular pressure
SO: Silicone oil
