# Supplementary material for: Macular edema after rhegmatogenous retinal detachment repair: risk factors, OCT analysis, and treatment responses
Source: Int J Retina Vitreous. 2021 Jan 25;7:9. doi: 10.1186/s40942-020-00254-9 (PMC7831177; doi:10.1186/s40942-020-00254-9)
Supplement: Supplementary file 1 — Additional file 1: Table S1. Descriptive statistics for all three groups, as explained in results population section. [file 40942_2020_254_MOESM1_ESM.docx]

|  | **tCME** | **cCME** | **nCME** | ***^1^P* Value** |
| --- | --- | --- | --- | --- |
| **Demographic Data** |  |  |  |  |
| **Number of eyes** | 5 (5%) | 20 (20%) | 74 (75%) |  |
| **Follow-up (months)** | 28.8 ± 14.2 | 19.6 ± 11.1 | 20.4 ± 10.8 | 0.33 |
| **Sex, Female** | 5 (100%) | 12 (60%) | 38 (34%) | 0.07 |
| **Age (years)** | 64.0 ± 7.4 | 64.1 ± 12.6 | 56.7 ± 18.0 | 0.24 |
| **Clinical Data** |  |  |  |  |
| **Right eye** | 2 (40%) | 9 (45%) | 40 (54%) | 0.70 |
| **Glaucoma** | 1 (20%) | 3 (15%) | 6 (8%) | 0.34 |
| **Lens status** |  |  |  | < 0.001 |
| Phakic | 1 (20%) | 0 (0%) | 44 (60%) |  |
| Pseudophakic | 3 (60%) | 11 (55%) | 28 (38%) |  |
| Aphakic | 1 (20%) | 9 (45%) | 2 (3%) |  |
| **Macula off^a^** | 3/4 (75%) | 17/20 (85%) | 31/70 (44%) | 0.001 |
| **PVR Stage C^a^** | 2/5 (40%) | 13/19 (68%) | 5/74 (7%) | < 0.001 |
| **Final VA (LogMAR)** | 0.18 ± 0.24 | 1.01 ± 0.80 | 0.20 ± 0.30 | < 0.001 |
| **ERM** | 2 (40%) | 17 (85%) | 28 (38%) | 0.001 |
| **Surgical details** |  |  |  |  |
| **Number of surgeries** | 1.8 ± 1.3 | 3.9 ± 1.7 | 1.4 ± 0.9 | < 0.001 |
| **Multiple PPV** | 2 (40%) | 19 (95%) | 17 (23%) | < 0.001 |
| **Referred after surgery elsewhere** | 0 (0%) | 12 (60%) | 5 (7%) | < 0.001 |
| **Number of surgery outside** |  | 1.25 ± 1.3 | 0.095 ± 0.4 | < 0.001 |
| **Type of surgery** |  |  |  | < 0.001 |
| *SB* | *1 (20%)* | *0 (0%)* | *25 (34%)* |  |
| *PPV* | *1 (20%)* | *6 (30%)* | *28 (38%)* |  |
| *PPV+SB* | *3 (60%)* | *14 (70%)* | *21 (28%)* |  |
| **Tamponade agent** |  |  |  | < 0.001 |
| *None/Air* | *1 (20%)* | *0 (0%)* | *24 (32%)* |  |
| *Gas (SF_6_ or C_3_F_8_)* | *3 (60%)* | *5 (25%)* | *46 (62%)* |  |
| *Silicone Oil* | *1 (20%)* | *15 (75%)* | *4 (5%)* |  |
| **Cryotherapy^a^** | 1/5 (20%) | 3/19 (16%) | 30/73 (41%) | 0.10 |
| **Retinectomy** | 0 (0%) | 9 (45%) | 4 (5%) | < 0.001 |
| **PFCL^a^** | 3/4 (75%) | 15/19 (79%) | 35/47 (75%) | 0.99 |

tCME: transient cystoid macular edema; cCME: chronic cystoid macular edema; nCME: no cystoid macular edema; PVR: proliferative vitreoretinopathy; VA: visual acuity; LogMAR: (logarithm of the minimum angle of resolution); PPV: pars-plana vitrectomy; ERM: epiretinal membrane; SB: scleral buckle; PFCL: perfluorocarbon liquid

^a^Denominators are provided if the number is less than the total number of eyes in the category due to missing or incomplete data

*^1^*From a Kruskall-Walis ANOVA or Fisher’s exact test.

Table S1: Demographics, Baseline Characteristics, and Surgical Data of Patients with tCME, cCME and nCME.
